# The Effect of Meditation and Physical Activity on the Mental Health Impact of COVID-19–Related Stress and Attention to News Among Mobile App Users in the United States: Cross-sectional Survey

**DOI:** 10.2196/28479

**Published:** 2021-04-13

**Authors:** Jennifer Green, Jennifer Huberty, Megan Puzia, Chad Stecher

**Affiliations:** 1 College of Health Solutions Arizona State University Phoenix, AZ United States; 2 Behavioral Research and Analytics, LLC Salt Lake City, UT United States

**Keywords:** coronavirus, health behavior, mindfulness meditation, mHealth, COVID-19, mental health

## Abstract

**Background:**

The COVID-19 pandemic has been declared an international public health emergency, and it may have long-lasting effects on people’s mental health. There is a need to identify effective health behaviors to mitigate the negative mental health impact of COVID-19.

**Objective:**

The objectives of this study were to (1) examine the regional differences in mental health and COVID-19–related worry, attention to news, and stress, in light of the state-level prevalence of COVID-19 cases; (2) estimate the associations between mental health and COVID-19–related worry, attention to news, and stress and health behavior engagement (ie, physical activity, mindfulness meditation); and (3) explore the mediating effect of health behavior engagement on the associations between mental health and COVID-19–related worry, attention to news, and stress.

**Methods:**

A cross-sectional survey was distributed to a sample of US adult paying subscribers to the Calm app (data were collected from April 22 to June 3, 2020). The survey assessed COVID-19–related worry, attention to news, and stress; health behavior engagement; and mental health (ie, perceived stress, posttraumatic stress disorder, and anxiety and depression). Statistical analyses were performed using R software. Differences in COVID-19–related worry, attention to news, and stress and mental health by location were assessed using *t* tests and chi-square tests. Logistic and ordinary least squares models were used to regress mental health and health behavior on COVID-19–related worry, attention to news, and stress; moreover, causal mediation analysis was used to estimate the significance of the mediation effects.

**Results:**

The median age of the respondents (N=8392) was 47 years (SD 13.8). Participants in the Mid-Atlantic region (New Jersey, New York, and Pennsylvania) reported higher levels of stress, more severe depression symptoms, greater worry about COVID-19, paying more attention to COVID-19–related news, and more stress related to social distancing recommendations than participants living in other regions. The association between worry about COVID-19 and perceived stress was significantly mediated by changes in physical activity (*P*<.001), strength of meditation habit (*P*<.001), and stopping meditation (*P*=.046). The association between worry about COVID-19 and posttraumatic stress disorder symptoms was significantly mediated by changes in physical activity (*P*<.001) and strength of meditation habit (*P*<.001).

**Conclusions:**

Our findings describe the mental health impact of COVID-19 and outline how continued participation in health behaviors such as physical activity and mindfulness meditation reduce worsening of mental health due to the COVID-19 pandemic. These data have important implications for public health agencies and health organizations to promote the maintenance of health habits to reduce the residual mental health burden of the COVID-19 pandemic.

## Introduction

In January 2020, the World Health Organization declared COVID-19 an international public health emergency [1], and the negative mental health effects of the ongoing COVID-19 pandemic are expected to be a significant, long-lasting global health problem [2,3]. In an April 2020 review of the existing literature on COVID-19 and mental health, moderate to severe levels of depressive symptoms and anxiety were reported in 16%-28% of the general population and medical staff in response to the COVID-19 pandemic [4]. Additionally, fear and worry about COVID-19 are common [5], with many people citing worries related to personal infection or the infection of family members [6,7], an overrun health care system, financial losses without expectation of recovery soon [8,9], and long-lasting isolation and movement restrictions [10]. Previously reported data have suggested regional differences across the United States in COVID-19–related fear and mental health (ie, anxiety and depressive symptoms), with greater symptoms in regions with higher confirmed cases, namely the Northeast New England, Northeast Mid-Atlantic, South-South Atlantic, and West Pacific regions (survey data collected March 23, 2020) [5]. Based on the known mental health effects of the COVID-19 pandemic, there is a clear need for strategies to help individuals better cope with the pandemic and mitigate its potentially long-lasting mental health consequences. Additionally, there is a need to better understand how resources for mental health should be allocated as the prevalence of COVID-19 infections changes regionally over time.

Although mental health professionals are often among the first line of treatment for poor mental health, digital approaches, including mobile health (mHealth) technologies may provide a way to more widely disseminate treatment information and enable individuals to self-manage their mental health from the safety of their own home. In a recent survey (N=2198) by the Academy of Medical Services, many respondents were concerned about how they would access mental health support, as many previously available in-person services had been discontinued as a result of the COVID-19 pandemic [11]. Stakeholders were also concerned with the capacity to handle the increased demand for mental health services and the lack of emphasis on mental health compared to treatment of COVID-19 and its physical health impacts [11]. Importantly, self-management strategies, including digital approaches to improve mental health, have become an area of interest for policy makers, as many individuals fail (or are unable) to participate due to the pandemic (eg, facility closures, reduced client load, social distancing) [12]. Self-management strategies may also empower individuals by enabling them to take a more active role in their health care (ie, recognizing and managing their own health problems) [13]. Additionally, self-management strategies are cost-effective and can be used as a preventative tool (rather than prescriptive or treatment-focused) that may mitigate the development of more debilitating mental health issues [12]. Common and evidence-based self-management strategies for improving mental health are physical activity and mindfulness meditation [14-16], both of which can still be maintained during social distancing policies and stay-at-home orders and can be adapted and delivered digitally. However, the extent to which individuals have maintained their participation in these self-management strategies during the COVID-19 pandemic is unknown.

Physical activity has been widely adopted as a beneficial way to self-manage mental health and may attenuate the mental health decline resulting from COVID-19 [17,18]. Physical activity has been shown to be as effective as antidepressants in decreasing stress, improving mood, and enhancing self-esteem [17,18]. Despite the known benefits of physical activity, currently, more than 60% of US adults do not engage in the recommended amount of physical activity (ie, 150 minutes of moderate to vigorous physical activity per week), and 25% are not active at all (ie, sedentary) [19]. Social distancing, quarantine or social isolation, and closure of public spaces due to COVID-19 are likely to increase these rates of physical inactivity [20,21], and the degree to which adults change the type or duration of their physical activity during COVID-19 is also unknown. Thus, there is a need to determine how self-management strategies such as physical activity have changed as a result of the pandemic, and specifically if reductions in physical activity are associated with worsening mental health status.

Mindfulness meditation, another self-management strategy, has also been evidenced to improve mental health, and maintenance (or habituation) of this behavior, particularly during the COVID-19 pandemic, may reduce worsening of mental health over time. Evidence suggests that mindfulness meditation reduces stress, improves mood (eg, symptoms of anxiety and depression), and enhances well-being [14,22]. Although an optimal amount of mindfulness meditation has not been established, evidence suggests that its benefits accrue with greater frequency of practice [23]. The habituation of mindfulness meditation practice (ie, behavioral automaticity) [24,25] has yet to be examined in the literature [26] but may have important implications for whether individuals maintain their practice during the COVID-19 pandemic. Those with stronger meditation habits may be more likely to continue meditation practice even when daily life is disrupted, as habits are known to persist when motivation declines and other distractions are present [25]. Mindfulness meditation delivered via mHealth may be another useful way to help people maintain their practice, especially when many public facilities are closed or limit participation [27-29], and popular meditation apps such as Calm or Headspace have shown promise to reduce stress and improve health [27,30-34]. Interestingly, sales of the Calm app in April 2020 were 62% higher than in February 2020 and 32% higher than in March 2020 (unpublished sales data provided by an internal Calm team), indicating that more people are accessing this type of self-management strategy in response to COVID-19. Although mHealth meditation apps have promise to help people self-manage their mental health, there is a lack of understanding about how continued participation in meditation is being impacted during the COVID-19 pandemic and whether meditation habits are associated with improved mental health.

Given the benefits of physical activity and mindfulness meditation on mental health, the purpose of this cross-sectional survey (data collected between April 22 and June 3, 2020) was to (1) examine the differences in COVID-19–related worry, attention to news, and stress from social distancing and mental health (ie, stress, posttraumatic stress disorder [PTSD], depression, and anxiety) by region of the United States, (2) explore the associations between COVID-19–related worry, attention to news, and stress and health behavior engagement (ie, strength of meditation habit, and changes in mindfulness meditation and physical activity) and mental health, and (3) estimate the mediating effect of health behavior engagement on the associations between mental health and COVID-19–related worry, attention to news, and stress. We hypothesized that (1) there would be differences in COVID-19–related worry, attention to news, and stress and mental health in states with higher prevalence of COVID-19, (2) greater COVID-19–related worry, attention to news, and stress would be associated with lower health behavior engagement and greater levels of mental health, and (3) health behavior engagement would mediate the associations between mental health and COVID-19–related worry, attention to news, and stress.

## Methods

### Ethics Approval

The Institutional Review Board at Arizona State University (STUDY00011867) approved the study. All participants provided electronic consent before participating in the study. The data sets generated and analyzed during the study are available at OSF [35].

### Study Design

This survey is part of a descriptive, national longitudinal study using a nonrandom convenience sample of adult paying subscribers to Calm, a mindfulness meditation mobile app. Participants in this study initially completed a cross-sectional baseline survey called the “COVID-19 Health and Well-being Survey” and agreed to complete four follow-up surveys over the subsequent 12 months. The data presented in this paper were obtained from the baseline survey administered from April 22 to June 3, 2020.

### Participant Recruitment and Selection

Emails inviting Calm subscribers to participate in the study were sent on April 22, April 29, and May 6, 2020. Subscribers were eligible if they had opened an email from Calm at least once in the last 90 days and had used Calm at least once in the last 90 days, were aged 18 years or older, could read and understand English, and resided in the United States.

### Procedures

Interested individuals were directed to a Qualtrics eligibility screener (~1 minute to complete). Once eligibility was determined, participants completed an electronic informed consent form and the baseline survey. There were no incentives for participation in the first wave (baseline) of the study; however, incentives were offered for continued participation in subsequent waves of this study (ie, random draws for 20 US $50 Amazon gift cards at months 2-4 and 50 US $50 Amazon gift cards at month 12).

### COVID-19 Health and Well-being Survey

The baseline survey was also administered using Qualtrics and included both investigator-developed and validated questionnaires. The investigator-developed portion of the survey included a total of 15 questions related to worry regarding COVID-19, attention to news, and stress from social distancing and 20 questions related to health behavior engagement (ie, strength of meditation habit and changes in mindfulness meditation and physical activity; see Textbox 1 for the questions used in these analyses). The COVID-19–related questions were adapted from a US Centers for Disease Control and Prevention (CDC) questionnaire for other infectious diseases (eg, the Severe Acute Respiratory Syndrome [SARS] Psychosocial Research Consortium survey) [36,37]. The validated survey components assessed meditation habit strength, perceived stress, PTSD, and anxiety and depression. Demographic information was collected at the end of the survey. Specifically, zip codes were used to determine the participants’ state of residence [38], and the states were categorized into regions according to the US Census Bureau classification system [39]. States were also designated as having a high or low prevalence of COVID-19 based on data compiled by the Center for Systems Science and Engineering at Johns Hopkins University describing state-level COVID-19 cases and deaths per 100,000 at the time of the initial survey distribution (April 22, 2020) [40].

**Investigator-developed survey questions used in the analyses.**

Prior to COVID did you meditate with Calm? [Y/N]

Do you currently meditate with Calm? [Y/N]

To what extent has COVID changed your meditation practice?

- I meditate much more (5)
- I meditate a little more (4)
- I meditate about the same amount (3)
- I meditate a little less (2)
- I meditate much less (1)
- I no longer meditate (0)

Prior to COVID, on average, how often did you engage in physical activity/exercise? [0-7 days/week]

On average, how often do you currently engage in physical activity/exercise? [0-7 days/week]

To what extent has COVID-19 changed your amount of physical activity/exercise?

- I exercise much more (5)
- I exercise a little more (4)
- I exercise about the same amount (3)
- I exercise a little less (2)
- I exercise much less (1)
- I no longer exercise (0)

Have recommendations for socially distancing caused stress for you?

- Not at all (1)
- A little (2)
- Somewhat (3)
- A lot (4)

How worried are you about…

… personally getting coronavirus?

… a family member getting coronavirus?

… the spread of coronavirus in your area?

- Not at all worried (1)
- A little worried (2)
- Somewhat worried (3)
- A good bit worried (4)
- Very worried (5)

How would you rate your attentiveness to information about ongoing changes and updates regarding the coronavirus?

- Not at all paying attention to (1)
- Somewhat paying attention to (2)
- Moderately paying attention to (3)
- Quite a bit paying attention to (4)
- Very much paying attention to (5)

### Measures

#### COVID-19–Related Worry, Attention to News, and Stress From Social Distancing

Participants were asked how worried they were about personally contracting COVID-19, a family member contracting COVID-19, and the spread of COVID-19 in their area (see Textbox 1). Worry about COVID-19 was operationalized as the sum of responses to these three questions. Scores ranged from 3-15, where higher scores indicate greater levels of worry about COVID-19. Participants were also asked how they would rate their attentiveness to information about ongoing changes and news regarding COVID-19 (see Textbox 1). Scores at or below the median were used to identify individuals with low attention to COVID-19, while scores above the median were categorized as high attention to COVID-19. Finally, the participants were asked if recommendations for socially distancing had caused them stress (see Textbox 1). Scores at or below the median were used to identify participants with low stress; scores above the median indicated high stress.

### Health Behavior Engagement

#### Mindfulness Meditation Practice

Participants were asked about their meditation practice using Calm prior to the COVID-19 pandemic as well as their current use of Calm. If participants indicated that they meditated using Calm prior to the COVID-19 pandemic, they were asked to what extent COVID-19 had changed their meditation practice (see Textbox 1). Participants who indicated they no longer participated in meditation were categorized as “stopped meditation,” while all other participants were considered as “continuing meditation.”

#### Physical Activity Behavior

Participants were asked to select how many days per week (scale of 0-7) they participated in physical activity prior to the COVID-19 pandemic as well as their current participation. Participants were also asked to what extent COVID-19 had changed the frequency or duration of their physical activity (see Textbox 1). Changes in physical activity were calculated as the difference between the participants’ reported number of days of physical activity currently and prior to the COVID-19 pandemic.

### Validated Surveys

#### Self-Report Habit Index

The Self-Report Habit Index (SRHI) includes 12 items reflecting on three proposed characteristics of habit (ie, automaticity, frequency, and relevance to self-identity). Response options range from 1, strongly disagree, to 5, strongly agree. The SRHI is a reliable and valid measure that has demonstrated Cronbach α values of .89-.92. The items are summed to produce a total score, with higher values indicating stronger habits [41]. For the current study, only the 4-item automaticity subscale of the SRHI was used in the analyses, and it produced a Cronbach α of .88.

#### Perceived Stress Scale

The Perceived Stress Scale (PSS) includes 10 items that measure the degree of self-appraised stress in one’s life within the past month [42,43]. The response items are rated on a 5-point Likert scale from 0, never, to 4, very often. The items are summed to produce a total score from 0-40, with higher scores indicating higher levels of perceived stress. The PSS is a reliable and valid measure that has demonstrated good internal consistency (Cronbach α=.74-.91) [44]. For the current study, the Cronbach α is .89.

#### Impact of Events Scale-6

The Impact of Events Scale-6 (IES-6) is a 6-item abbreviated version of the Impact of Events Scale-Revised (22 items) that assesses PTSD symptoms over the past seven days [45]. The response items are rated on a 5-point Likert scale from 0, not at all, to 4, extremely. The score is calculated as a mean of the six items. Scores range from 0-5, with a binary cutoff score of 1.75 indicating clinically important PTSD symptoms. The IES-6 is a valid and reliable measure with excellent internal consistency (α=.91). For the current study, the Cronbach α is .86.

#### Hospital Anxiety and Depression Scale

The Hospital Anxiety and Depression Scale (HADS) is a 14-item scale measuring levels of anxiety and depression [46]. The anxiety subscale (HADS-A) and the depression subscale (HADS-D) each comprise 7 items. Response items are rated on a 4-point Likert scale from 0-3. The items are summed to produce a total score from 0-21 on each subscale. Scores from 0-7 are considered normal, scores from 8-10 are considered borderline abnormal, and scores from 11-21 are considered abnormal. The HADS is a valid and reliable tool, with internal consistencies reported to be as high as α=0.93 and α=0.90 for the HADS-A and HADS-D subscales, respectively. For the current study, the Cronbach α=.85 and .80 for the HADS-A and HADS-D, respectively.

### Statistical Analysis

All statistical analyses were performed using R software, version 4.0.0 (R Project) [47]. Descriptive statistics were used to characterize the sample’s demographic characteristics; health; presence of chronic conditions; mental health; and COVID-19–related worry, attention to news, and stress. Differences in mental health and COVID-19–related worry, attention to news, and stress by location were assessed using *t* tests and chi-square tests. Logistic and ordinary least squares models were used to regress mental health and health behavior on COVID-19–related worry, attention to news, and stress, and causal mediation analysis from the “mediation” package in R was used to estimate the significance of the mediation effects. Specifically, the standard errors for the mediation effects were calculated from 1000 bootstrapped samples for each mediation regression. Demographic, health, and location variables were included as covariates in all regression analyses. A *P* value of <.05 was considered statistically significant.

## Results

### Sample Characteristics

The sample (N=8392) was primarily White, non-Hispanic, and female (see Table 1). The majority of participants had a bachelor’s or graduate-level degree, were employed, and had an annual household income exceeding US $100,000. Approximately one-third of the participants (2603/7335, 35.49) reported having a least one medical condition associated with increased risk of severe illness from COVID-19; however, more than 80% (5976/7317, 81.67%) perceived themselves to be in good overall health.

The self-reported changes in preventative health behaviors are shown in Table 2; most participants were found to have increased or maintained their physical activity and meditation habits during the initial period of the COVID-19 pandemic. There was a significant correlation between strength of meditation habit and changes in meditation during the COVID-19 pandemic; participants with the strongest habits were the most likely to increase or maintain their meditation practices (*r*=0.37, *P*<.001).

The mental health characteristics of the sample at the time of the baseline survey are presented in Table 3. Compared to participants living in other parts of the country, participants living in the Mid-Atlantic region (ie, New Jersey, New York, or Pennsylvania) reported higher levels of stress and more severe depression symptoms. Participants living in the South Atlantic region (ie, Delaware, Florida, Georgia, Maryland, North Carolina, South Carolina, Virginia, West Virginia, or District of Columbia) reported less severe depressive symptoms than participants in other regions. Participants living in states with a high prevalence of COVID-19 cases (ie, California, Colorado, Illinois, Massachusetts, New Jersey, New York, or Washington) reported more severe PTSD symptoms than participants living in states where COVID-19 was less prevalent.

Table 4 presents the participants’ reports regarding their worries about COVID-19 (ie, contracting it themselves, a family member contracting it, the spread in their area), their attentiveness to news and updates regarding COVID-19, and their stress due to COVID-19–related social distancing recommendations. Participants in the Mid-Atlantic region were more worried about COVID-19, paid more attention to COVID-19 news and updates, and reported more stress related to social distancing recommendations than participants living in other regions. Conversely, participants living in the South Atlantic region reported less stress from social distancing recommendations than participants in other regions. Participants living in states where COVID-19 was prevalent had more COVID-19–related worry, paid more attention to COVID-19 news and updates, and experienced more stress from social distancing recommendations.

The primary sources participants used to acquire information about COVID-19 were news media (eg, newspapers, web-based newspapers, television news networks; n=5552/7371, 75.32%) and health officials (eg, CDC, World Health Organization, state health officials; n=5212/7371, 75.12%). Approximately one-third of participants reported acquiring information about COVID-19 from social media (n=2951/7371, 40.04%), doctors and medical professionals (n=2721/7371, 36.91%), and friends and family members (n=2543/7371, 34.50%).

**Table 1:** Demographic and health characteristics of the sample (N=8392).

Characteristic			Value
Age (years), median (SD)			47.0 (13.8)
**Gender (n=7303), n (%)**			
	Female	6129 (83.92)	
	Male	1147 (15.71)	
	Other	27 (0.37)	
**Race (n=7178), n (%)**			
	White	6586 (91.75)	
	Black/African American	231 (3.22)	
	Asian	216 (3.01)	
	Native American/Alaska Native	83 (1.16)	
	Native Hawaiian/Pacific Islander	27 (0.38)	
	Other	195 (2.72)	
**Ethnicity (n=6774), n (%)**			
	Non-Hispanic	6338 (93.56)	
	Hispanic	436 (6.44)	
People in the household, median (SD)			2.0 (1.4)
**Income (US $; n=6949), n (%)**			
	20,000 or less	212 (3.05)	
	21,000-40,000	402 (5.79)	
	41,000-60,000	705 (10.15)	
	61,000-80,000	942 (13.56)	
	81,000-100,000	1055 (15.18)	
	More than 100,000	3633 (52.28)	
**Employment (n=7297), n (%)**			
	Employed	5084 (69.67)	
	Retired	1012 (13.87)	
	Unemployed	477 (6.54)	
	Homemaker	306 (4.19)	
	Unable to work	252 (3.45)	
	Student	166 (2.27)	
**Education (n=7319), n (%)**			
	11th grade or less	8 (0.11)	
	High school or General Educational Development	161 (2.20)	
	Some college	826 (11.29)	
	Two-year/technical degree	424 (5.79)	
	Bachelor's degree	2670 (36.48)	
	Graduate degree	3230 (44.13)	
**Region^a^ (n=7037), n (%)**			
	New England	489 (6.95)	
	Mid-Atlantic	946 (13.44)	
	East North Central	953 (13.54)	
	West North Central	459 (6.52)	
	South Atlantic	1192 (16.94)	
	East South Central	198 (2.81)	
	West South Central	522 (7.42)	
	Mountain West	713 (10.13)	
	Pacific West	1565 (22.24)	
**State COVID-19 prevalence^b^ (n=7037), n (%)**			
	High-prevalence state	3062 (43.51)	
	Low-prevalence state	3975 (56.49)	
**Health rating (n=7317), n (%)**			
	Poor	1341 (18.33)	
	Good	5976 (81.67)	
**Underlying medical conditions^c^ associated with increased risk of severe illness (n=7335), n (%)**			
	At least one underlying medical condition	2603 (35.49)	
	No underlying medical conditions	4732 (64.51)	

^a^States within each region are based on US Census divisions.

^b^State-level COVID-19 prevalence is based on the number of COVID-19 cases per 100,000 at the time of the first survey distribution (April 22, 2020); high-prevalence states were California, Colorado, Illinois, Massachusetts, New Jersey, New York, and Washington.

^c^The underlying medical conditions associated with increased risk of severe illness from COVID-19 include asthma, cardiovascular disease, chronic lung disease, diabetes, chronic kidney disease, cancer in the past year, immunosuppressant therapy, and hepatitis B.

**Table 2:** Self-reported engagement in preventative health behaviors (N=8392).

Behavior			Value
**Physical activity prevalence (N=7325), n (%)**			
	Physical activity performance prior to the COVID-19 pandemic	6715 (91.67)	
	Physical activity performance since the COVID-19 pandemic	6468 (88.30)	
**Physical activity change since the COVID-19 pandemic (n=6015), n (%)**			
	Increased	1394 (23.18)	
	Maintained	1585 (26.35)	
	Decreased	2537 (42.18)	
	Stopped	499 (8.30)	
**Meditation prevalence (n=7332), n (%)**			
	Meditated prior to the COVID-19 pandemic	5940 (81.01)	
	Meditated since the COVID-19 pandemic	5435 (74.13)	
**Medication change since the COVID-19 pandemic (n=5914), n (%)**			
	Increased	2101 (35.53)	
	Maintained	1979 (33.46)	
	Decreased	1479 (25.01)	
	Stopped	355 (6.00)	
Strength of meditation habit (SRHI^a^ score), mean (SD)			10.72 (4.05)

^a^SRHI: Self-Report Habit Index.

**Table 3:** Differences in mental health by region and state-level COVID-19 prevalence.

Characteristic		Stress^a^			PTSD^b,c^			Depression^d^			Anxiety^e^	
		Mean (SD)	*P* value	Mean (SD)		*P* value	Mean (SD)		*P* value	Mean (SD)		*P* value
**Region^f^**												
	New England	18.31 (6.47)	.37	1.68 (0.89)		.82	8.92 (4.15)		.70	6.01 (3.75)		.36
	Mid-Atlantic	18.66 (6.46)	<.001	1.72 (0.91)		.62	9.13 (4.22)		.03	6.07 (3.81)		.07
	East North Central	17.75 (6.33)	.12	1.66 (0.90)		.56	8.81 (4.20)		.71	5.77 (3.62)		.40
	West North Central	17.92 (6.29)	.65	1.65 (0.87)		.56	8.86 (4.09)		.97	5.97 (3.82)		.51
	South Atlantic	17.81 (6.31)	.14	1.63 (0.87)		.06	8.51 (4.04)		<.001	5.73 (3.57)		.18
	East South Central	18.76 (6.87)	.14	1.67 (0.93)		.92	9.15 (4.43)		.34	5.92 (3.70)		.81
	West South Central	17.73 (6.43)	.24	1.62 (0.92)		.14	8.86 (4.24)		.96	5.67 (3.63)		.22
	Mountain West	18.03 (6.13)	.94	1.69 (0.86)		.56	8.94 (4.10)		.54	5.82 (3.47)		.73
	Pacific West	18.03 (6.14)	.88	1.70 (0.87)		.16	8.88 (4.06)		.79	5.88 (3.50)		.79
**State COVID-19 prevalence^g^**												
	High-prevalence state	18.23 (6.21)	.08	1.71 (0.86)		.004	8.89 (4.06)		.80	5.92 (3.60)		.20
	Low-prevalence state	17.97 (6.38)	N/A^h^	1.65 (0.89)		N/A	8.86 (4.18)		N/A	5.81 (3.64)		N/A
Total, median (SD)		18.08 (6.31)	N/A	1.67 (0.89)		N/A	8.72 (4.13)		N/A	5.86 (3.62)		N/A

^a^Stress was measured using the total score on the Perceived Stress Scale.

^b^PTSD: posttraumatic stress disorder.

^c^PTSD was measured using mean scores on the Impact of Events Scale-6.

^d^Depression was measured using the Depression subscale score on the Hospital Depression and Anxiety Scale.

^e^Anxiety was measured using the Anxiety subscale score on the Hospital Depression and Anxiety Scale.

^f^States within each region are based on US Census divisions.

^g^State-level COVID-19 prevalence is based on the number of COVID-19 cases per 100,000 at the time of the first survey distribution (April 22, 2020); high-prevalence states were California, Colorado, Illinois, Massachusetts, New Jersey, New York, and Washington.

^h^N/A: not applicable.

**Table 4:** Differences in COVID-19–related worry, attention to news, and stress by region and state-level COVID-19 prevalence.

Variable		Worry			High attention to news			High stress from social distancing		
		Mean (SD)	*P* value	n (%)		*P* value	n (%)		*P* value	
**Region**										
	New England	9.69 (2.85)	.54	155 (31.70)		.36	233 (47.65)		.12	
	Mid-Atlantic	10.11 (2.86)	<.001	353 (37.35)		.01	468 (49.47)		<.001	
	East North Central	9.58 (2.94)	.70	312 (32.74)		.53	404 (42.44)		.26	
	West North Central	9.55 (2.78)	.61	153 (33.33)		.91	213 (46.41)		.35	
	South Atlantic	9.59 (2.92)	.74	395 (33.14)		.69	494 (41.44)		.39	
	East South Central	9.75 (3.01)	.53	69 (34.85)		.78	82 (41.41)		.47	
	West South Central	9.32 (3.08)	.03	162 (31.03)		.20	209 (40.04)		.053	
	Mountain West	9.41 (2.99)	.052	225 (31.56)		.22	300 (42.08)		.25	
	Pacific West	9.52 (2.90)	.16	544 (34.85)		.28	706 (45.11)		.42	
**State COVID-19 prevalence^a^**										
	High-prevalence state	9.72 (2.90)	.01	1064 (34.81)		.04	1420 (46.37)		.004	
	Low-prevalence state	9.53 (2.95)	N/A^b^	1387 (32.51)		N/A	1832 (42.92)		N/A	
Total		9.61 (2.93)	N/A	2451 (33.47)		N/A	3252 (44.37)		N/A	

^a^State-level COVID-19 prevalence is based on the number of COVID-19 cases per 100,000 at the time of the baseline survey (April 22, 2020); high-prevalence states were California, Colorado, Illinois, Massachusetts, New Jersey, New York, and Washington.

^b^N/A: not applicable.

### Associations Between Health Behavior Engagement and COVID-19–Related Worry, Attention to News, and Stress

Participants who were more worried about COVID-19, paid more attention to COVID-19 news and updates, and experienced more stress due to COVID-19 social distancing recommendations had greater decreases in physical activity and lower strength of their meditation habits (see Table 5). Attention to news and updates about COVID-19 and stress due to social distancing recommendations were also associated with stopping meditation.

Men, White and non-Hispanic respondents, and respondents with higher levels of education and higher household incomes were also less likely to decrease their engagement in physical activity. Strength of meditation habit was also generally greater among respondents who were older, White, non-Hispanic, female, and more educated, and who had higher annual household incomes. Younger participants and men were more likely to report that since the COVID-19 pandemic, they had stopped meditating. Living in a state with high COVID-19 prevalence was associated with decreases in physical activity and lower strength of meditation habit but not with stopping meditation practice.

**Table 5:** Associations between health behavior engagement and COVID-19–related worry, attention to news, and stress. The table presents the coefficients from ordinary least squares regression models for the continuous outcomes, changes in physical activity, and meditation habit strength on COVID-19–related worry, attention to news, and stress, and logistic regression models for the stopped meditation outcome on COVID-19–related worry, attention to news, and stress. Standard errors were estimated using heteroscedasticity-robust procedures.

Outcome and covariates				COVID-19 worry			COVID-19 attention			Stress about social distancing	
			Coefficient (SE)		*P* value	Coefficient (SE)		*P* value	Coefficient (SE)		*P* value
**Outcome: changes in physical activity**											
	COVID-19 worry		–0.04 (0.01)		<.001	—^a^		—	—		—
	COVID-19 attention		—		—	–0.16 (0.06)		.01	—		—
	Stress about social distancing		—		—	—		—	–0.16 (0.05)		.003
	**Demographic covariates^b^**										
		Age	–0.01 (0.002)		.01	–0.003 (0.002)		.09	–0.005 (0.002)		.02
		Racial minority status	0.02 (0.10)		.81	0.05 (0.10)		.60	0.05 (0.10)		.60
		Female	0.25 (0.07)		<.001	0.22 (0.07)		.002	0.24 (0.07)		.001
		Hispanic	–0.20 (0.11)		.07	–0.26 (0.11)		.02	–0.25 (0.11)		.03
		High school education only	–0.71 (0.19)		<.001	–0.70 (0.19)		<.001	–0.69 (0.19)		<.001
		Undergraduate education	–0.22 (0.06)		<.001	–0.22 (0.06)		<.001	–0.22 (0.06)		<.001
		Income <US $80,000	–0.29 (0.07)		<.001	–0.29 (0.07)		<.001	–0.28 (0.07)		<.001
		Income of US $81,000-100,000	–0.16 (0.06)		.01	–0.17 (0.06)		.01	–0.16 (0.06)		.01
		Unemployed	–0.08 (0.09)		.40	–0.07 (0.09)		.42	–0.07 (0.09)		.46
		Underlying medical condition	–0.12 (0.06)		.03	–0.15 (0.06)		.01	–0.16 (0.06)		.01
		Living in a state with high COVID-19 prevalence^c^	–0.01 (0.05)		.04	–0.11 (0.054)		.03	–0.12 (0.05)		.03
**Outcome:** **stopped meditation**											
	COVID-19 worry		0.04 (0.02)		.045	—		—	—		—
	COVID-19 attention		—		—	0.36 (0.13)		.004	—		—
	Stress about social distancing		—		—	—		—	0.31 (0.12)		.01
**Outcome:** **strength of meditation habit**											
	COVID-19 worry		–0.06 (0.02)		.001	—		—	—		—
	COVID-19 attention		—		—	–0.17 (0.12)		.18	—		—
	Stress about social distancing		—		—	—		—	–0.45 (0.11)		<.001

^a—^: not applicable.

^b^These covariates were included in all the models.

^c^State-level COVID-19 prevalence is based on the number of COVID-19 cases per 100,000 at the time of the first survey distribution (April 22, 2020); high-prevalence states were California, Colorado, Illinois, Massachusetts, New Jersey, New York, and Washington.

### Mediation Models

#### Perceived Stress and COVID-19–Related Worry, Attention to News, and Stress via Engagement in Health Behaviors

The mediating effect of health behavior changes on the associations between stress and COVID-19–related worry, attention to news, and stress from social distancing is demonstrated by the regression analyses presented in Table 6. The first row and first column of Table 6 present the total effect of worry about COVID-19 on self-reported stress, and the subsequent columns illustrate how the association between worry about COVID-19 and stress is attenuated by the inclusion of each behavioral change measure. Based on bootstrapped estimation procedures, we found that the association between worry about COVID-19 and perceived stress was significantly mediated by changes in physical activity (*P*<.001), stopping meditation (*P*=.046), and strength of meditation habit (*P*<.001). The second panel of Table 6 presents a similar mediation analysis for the association between attention to COVID-19 news and updates and perceived stress. The bootstrapped standard error calculations found that changes in physical activity (*P*=.02) and stopping meditation (*P*=.002) partially mediated the association between attention to COVID-19 news and updates and perceived stress; however, strength of meditation habit was not a significant mediator (*P*=.11). Finally, the association between perceived stress and stress due to COVID-19 social distancing recommendations was significantly mediated by changes in physical activity (*P*=.01), stopping meditation (*P*=.02), and strength of meditation habit (*P*<.001).

**Table 6:** Mediating effect of health behavior change on stress. The table presents the coefficients from ordinary least squares regression models of PSS stress score on COVID-19 worry, COVID-19 attention, and stress from social distancing as well as the indicated health behavior changes. The demographic variables of age, male sex, Hispanic, income <US $80,000, income US $81,000-100,000, unemployed, and underlying medical condition were included as covariates in all models, which also estimated heteroscedasticity-robust standard errors.

Variable			PSS^a^ stress score																				
		Total effect					Change in physical activity							Stopped meditating						Strength of meditation habit			
		Coefficient (SE)		*P* value		Coefficient (SE)			*P* value			Coefficient (SE)			*P* value			Coefficient (SE)			*P* value		
COVID-19 worry—total effect			0.61 (0.02)		<.001		—^b^			—			—			—			—			—	
**COVID-19 worry—indirect effect**								0.60 (0.03)			<.001			0.64 (0.028)			<.001			0.63 (0.03)			<.001
	Change in physical activity	—		—		–0.25 (0.03)			<.001			—			—			—			—		
	Stopped meditating	—		—		—			—			2.00 (0.33)			<.001			—			—		
	Strength of meditation habit	—		—		—			—			—			—			–0.15 (0.02)			<.001		
COVID-19 attention—total effect			0.77 (0.16)		<.001		—			—			—			—			—			—	
**COVID-19 attention—indirect effect**								0.74 (0.16)			<.001			0.65 (0.18)			<.001			0.76 (0.18)			<.001
	Change in physical activity	—		—		–0.30 (0.04)			<.001			—			—			—			—		
	Stopped meditating	—		—		—			—			2.15 (0.35)			<.001			—			—		
	Strength of meditation habit	—		—		—			—			—			—			–0.17 (0.02)			<.001		
Stress from social distancing—total effect			3.59 (0.16)		<.001		—			—			—			—			—			—	
**Stress from social distancing—indirect effect**								3.54 (0.14)			<.001			3.64 (0.16)			<.001			3.59 (0.16)			<.001
	Change in physical activity	—		—		–0.27 (0.03)			<.001			—			—			—			—		
	Stopped meditating	—		—		—			—			1.93 (0.33)			<.001			—			—		
	Strength of meditation habit	—		—		—			—			—			—			–0.15 (0.02)			<.001		

^a^PSS: Perceived Stress Scale.

^b^—: not applicable.

#### PTSD Symptoms and COVID-19–Related Worry, Attention to News, and Stress via Engagement in Health Behaviors

The mediating effect of health behavior changes on the associations between PTSD symptoms and COVID-19–related worry, attention to news, and stress is outlined by the regression analyses presented in Table 7. The first row and first column of Table 7 present the total effect of worry about COVID-19 on self-reported PTSD symptoms, and the subsequent columns illustrate how the association between worry about COVID-19 and PTSD symptoms is attenuated by the inclusion of each behavioral change measure. Based on bootstrapped estimation procedures, we found that the association between worry about COVID-19 and PTSD symptoms was significantly mediated by changes in physical activity (*P*<.001) and strength of meditation habit (*P*<.001) but not significantly mediated by stopping meditation (*P*=.07). The second panel of Table 7 presents the same mediation analysis for the association between attention to COVID-19 news and updates and PTSD symptoms, where bootstrapped standard error calculations indicated that changes in physical activity (*P*=.01) and stopping meditation (*P*=.004) significantly mediated the association between PTSD symptoms and attention to COVID-19 news and updates while the strength of meditation habit did not (*P*=.13). Finally, the association between PTSD symptoms and stress caused by COVID-19 social distancing recommendations was partially mediated by changes in physical activity (*P*=.01), stopping meditation (*P*=.01), and strength of meditation habit (*P*<.001).

**Table 7:** Mediating effect of health behavior change on PTSD symptoms. This table presents the coefficients from ordinary least squares regression models of PTSD score on COVID-19–related worry, attention to news, and stress from social distancing as well as the indicated health behavior changes. Age, male sex, Hispanic race, income <US $80,000, income US $81,000-100,000, unemployed, and underlying medical condition were included as covariates in all models.

Variable		PTSD^a,b^ score														
		Total effect			Change in physical activity				Stopped meditating					Strength of meditation habit		
		Coefficient (SE)	*P* value	Coefficient (SE)		*P* value		Coefficient (SE)		*P* value		Coefficient (SE)			*P* value	
COVID-19 worry—total effect		0.13 (0.003)	<.001	—^c^		—		—		—		—			—	
**COVID-19 worry—indirect effect**					0.13 (0.003)		<.001		0.13 (0.00)		<.001		0.13 (0.004)			<.001
	Change in physical activity	—	—	–0.03 (0.010)		<.001		—		—		—			—	
	Stopped meditating	—	—	—		—		0.23 (0.05)		<.001					—	
	Strength of meditation habit	—	—	—		—		—		—		–0.01 (0.003)			<.001	
COVID-19 attention—total effect		0.26 (0.02)	<.001	—		—		—		—		—			—	
**COVID-19 attention—indirect effect**					0.26 (0.02)		<.001		0.24 (0.03)		<.001		0.25 (0.03)			<.001
	Change in physical activity	—	—	–0.03 (0.010)		<.001		—		—		—			—	
	Stopped meditating	—	—	—		—		0.25 (0.05)		<.001		—			—	
	Strength of meditation habit	—	—	—		—		—		—		–0.02 (0.003)			<.001	
Stress from social distancing—total effect		0.55 (0.02)	<.001	—		—		—		—		—			—	
**Stress from social distancing‑indirect effect**					0.55 (0.02)		<.001		0.57 (0.02)		<.001		0.56 (0.02)			<.001
	Change in physical activity	—	—	–0.03 (0.010)		<.001		—		—		—			—	
	Stopped meditating	—	—	—		—		0.23 (0.05)		<.001		—			—	
	Strength of meditation habit	—	—	—		—		—		—		–0.01 (0.003)			<.001	

^a^PTSD: posttraumatic stress disorder.

^b^PTSD was measured using mean scores on the Impact of Events Scale-6.

^c^—: not applicable.

## Discussion

Our findings describe the mental health impact of the COVID-19 pandemic and outline how continued participation in health behaviors such as physical activity and mindfulness meditation reduce worsening of mental health due to the COVID-19 pandemic. The aim of this baseline survey was to first examine the regional differences in mental health and COVID-19–related worry, attention to news, and stress in light of the state-level prevalence of COVID-19 infections at the time of this survey. We additionally sought to estimate the associations between COVID-19–related worry and attention to news and stress, health behavior engagement, and mental health, as well as to explore the mediating effect of health behavior engagement on the associations between mental health and COVID-19–related worry, attention to news, and stress.

### Mental Health and COVID-19–Related Worry, Attention to News, and Stress by Region

Our findings indicate that participants living in the Mid-Atlantic region (ie, New Jersey, New York, or Pennsylvania) had higher levels of stress and more severe depressive symptoms compared to the rest of the country, which is consistent with the fact that a high prevalence of confirmed COVID-19 cases existed in this region during the time period when this survey was administered. Participants in the Mid-Atlantic region were also more worried about COVID-19, paid more attention to COVID-19 news and updates, and reported more stress related to social distancing recommendations than survey participants living in other regions. Additionally, participants living in states with a high prevalence of COVID-19 cases (ie, California, Colorado, Illinois, Massachusetts, New Jersey, New York, or Washington) reported more severe PTSD symptoms than those living in states where COVID-19 was less prevalent, and they also reported more COVID-19–related worry, paid more attention to COVID-19 news and updates, and experienced more stress from social distancing recommendations. These preliminary findings highlight the regional differences in worry and fear about COVID-19 and mental health based on the prevalence of COVID-19 cases. As the prevalence of COVID-19 cases among regions may change over time, this information could be particularly useful for public health agencies or community health centers to direct or provide more mental health resources to regions with high COVID-19 prevalence in an effort to mitigate the subsequent mental health burden. Additionally, future research and public health interventions should consider the local prevalence of COVID-19 and the extent to which individuals are paying attention to news and media to monitor news related to COVID-19 when targeting mental health interventions, as these factors may play an important role in the mental health impact of the pandemic.

### Associations of Worry About COVID-19, Physical Activity, Meditation, and Mental Health

Our findings suggest that higher levels of worry about COVID-19 were associated with lower physical activity levels and lower strength of meditation habits; however, overall, the majority of participants reported increasing or maintaining their physical activity or meditation practice. Although our findings are not causal, higher levels of worry about COVID-19 may have contributed to the difficulty people had in sustaining their physical activity participation and engaging in habitual meditation practice. This is aligned with other research demonstrating that anxiety and worry can be detrimental to habitual participation in health-promoting behaviors, both in general [48] and in the context of COVID-19 [49,50]. One cross-sectional survey conducted in Belgium during the COVID-19 lockdown reported increases in physical activity, but only in those who were previously less active; decreases in physical activity were found in previously highly active adults [51]. Conversely, other cross-sectional studies conducted during the COVID-19 pandemic reported significant declines in physical activity and increased sitting time [49,52,53]. No studies have reported patterns of meditation practice; however, one cross-sectional study assessing stress-coping behaviors conducted among New York City–based health care workers during the COVID-19 pandemic reported that meditation (23%) was a commonly endorsed behavior, along with physical activity/exercise (59%) [54]. However, the physical activity and meditation patterns were not assessed prior to the COVID-19 pandemic. More research describing patterns of health behaviors during the COVID-19 pandemic is needed to better understand how COVID-19 may contribute to long-lasting, negative health behavior changes.

Our data also suggest that higher levels of worry about COVID-19 are associated with poor mental health (ie, increased stress and PTSD), and importantly, this association between worry about COVID-19 and poor mental health was mediated by lower physical activity levels and lower strength of meditation habits (similar associations were observed for depression and anxiety; see Multimedia Appendix 1). Therefore, stopping or reducing health promoting behaviors, particularly physical activity and meditation practice, during the COVID-19 pandemic may increase the negative impact of COVID-19–related worry on stress, PTSD, depression, and anxiety. To our knowledge, this is the first study to examine the mediating effects of physical activity and meditation on the associations between worry about COVID-19 and mental health during the pandemic. Existing research has shown that greater concern about COVID-19 was associated with greater anxiety and depression levels [55], and decreased physical activity and increased sedentary time during COVID-19 was associated with poor mental health (ie, higher stress, anxiety, and depression symptoms) [49,56]. Additionally, both physical activity and meditation have been recommended as healthy ways to cope with stress during the COVID-19 pandemic, underscoring the importance of maintaining these behaviors during a time of heightened stress [57]. Our data demonstrate that participation in physical activity and meditation are important mechanisms in reducing the increased mental health problems associated with COVID-19–related worry. Other studies have shown that both mindfulness-based and physical activity interventions may be protective against the development of trauma-related psychopathology (eg, PTSD) by enhancing cognitive function, reducing arousal, normalizing hypothalamic pituitary axis function, and reducing inflammatory markers [58,59]. There is a need for more research, especially longitudinal data, to examine the long-term changes in health behaviors due to the COVID-19 pandemic, better disentangle the causal relationships between these psychological and behavioral outcomes, and identify strategies for helping people maintain health-promoting behaviors.

In summary, our findings support existing evidence of the beneficial health effects of physical activity and meditation on mental health outcomes [16,60-62]. Because the COVID-19 pandemic has profoundly impacted daily routines (eg, social distancing, quarantine, businesses closures) and may negatively impact the performance of health behaviors, it is important to continue promoting self-management of health behaviors such as physical activity and meditation that can reduce worsening of mental health during the COVID-19 pandemic, particularly in regions with a heightened sense of worry about COVID-19. Encouraging participation in physical activity and meditation should be an important public health objective during the current COVID-19 pandemic, especially because reduced physical health and poor mental health have been shown to increase susceptibility to COVID-19 infection and disease transmission [63,64]. Public health agencies may consider providing strategies to help people maintain or adapt their current health behaviors. For example, digital or mHealth interventions for both physical activity and meditation have shown promise for their feasibility, scalability, and physical and mental health benefits [64-67]. Digital and mHealth interventions are also convenient and often budget-friendly ways to encourage participation in both physical activity and meditation, and more research is needed to better understand their efficacy and applicability during the COVID-19 pandemic.

### Limitations

Although this study is one of the first to describe the associations between COVID-19–related worry and attention to news and stress, mental health, and self-management health behaviors, there are important limitations to be noted. First, our sample was primarily female, non-Hispanic, White, high-income, and highly educated, and the participants were paid subscribers of Calm, which limits the generalizability of these data. Second, this survey was cross-sectional; therefore, causal relationships cannot be determined from these analyses. In the broader study, we plan to implement four more surveys over the next 12 months to provide a more comprehensive longitudinal assessment of the impact of the COVID-19 pandemic on mental health and health behaviors.

### Conclusions

Our findings underscore the importance of maintaining self-management health behaviors such as physical activity and meditation for sustaining one’s mental health during the COVID-19 pandemic. These results suggest that public health agencies and health organizations should promote the maintenance of health habits with strategies such as digital and mHealth approaches that can be more easily adapted during stay-at-home orders and social distancing mandates. Future research is needed to identify the causal relationships between these psychological and behavioral outcomes and to evaluate strategies for helping people adapt their current meditation and physical activity practices to the restrictions on daily life imposed by COVID-19–related public health policies.

## Appendix

Multimedia Appendix 1 Supplementary tables.

## Abbreviations

CDC: US Centers for Disease Control and Prevention
HADS: Hospital Anxiety and Depression Scale
HADS-A: Hospital Anxiety and Depression Scale, anxiety subscale
HADS-D: Hospital Anxiety and Depression Scale, depression subscale
IES-6: Impact of Events Scale-6
mHealth: mobile health
PSS: Perceived Stress Scale
PTSD: posttraumatic stress disorder
SARS: severe acute respiratory syndrome
SRHI: Self-Report Habit Index

## References

[1] (2020). Mental health and psychosocial considerations during the COVID-19 outbreak. World Health Organization.

[2] Fiorillo A, Gorwood P (2020). The consequences of the COVID-19 pandemic on mental health and implications for clinical practice. Eur Psychiatry.

[3] Bao Y, Sun Y, Meng S, Shi J, Lu L (2020). 2019-nCoV epidemic: address mental health care to empower society. Lancet.

[4] Rajkumar RP (2020). COVID-19 and mental health: A review of the existing literature. Asian J Psychiatr.

[5] Fitzpatrick KM, Harris C, Drawve G (2020). Fear of COVID-19 and the mental health consequences in America. Psychol Trauma.

[6] Wang C, Pan R, Wan X, Tan Y, Xu L, Ho CS, Ho RC (2020). Immediate psychological responses and associated factors during the initial stage of the 2019 coronavirus disease (COVID-19) epidemic among the general population in China. Int J Environ Res Public Health.

[7] Brooks SK, Webster RK, Smith LE, Woodland L, Wessely S, Greenberg N, Rubin GJ (2020). The psychological impact of quarantine and how to reduce it: rapid review of the evidence. Lancet.

[8] Salisbury H (2020). Helen Salisbury: Fear in the time of covid. BMJ.

[9] Thombs B, Tao L, Wu Y, Levis B, Sun Y, Bourgeault A, Bhandari P, Jimenez A, Gagarine M, Harb S, He Chen, Krishnan A, Negeri ZF, Neupane D, Carrier ME, Kwakkenbos L, Benedetti A Preliminary COVID-19 Fears Questionnaire: systemic sclerosis and chronic medical conditions versions. OSF Preprints..

[10] Thombs BD, Bonardi O, Rice DB, Boruff JT, Azar M, He C, Markham S, Sun Y, Wu Y, Krishnan A, Thombs-Vite I, Benedetti A (2020). Curating evidence on mental health during COVID-19: a living systematic review. J Psychosom Res.

[11] Holmes EA, O'Connor RC, Perry VH, Tracey I, Wessely S, Arseneault L, Ballard C, Christensen H, Cohen Silver R, Everall I, Ford T, John A, Kabir T, King K, Madan I, Michie S, Przybylski AK, Shafran R, Sweeney A, Worthman CM, Yardley L, Cowan K, Cope C, Hotopf M, Bullmore E (2020). Multidisciplinary research priorities for the COVID-19 pandemic: a call for action for mental health science. Lancet Psychiatry.

[12] Inkster B, O'Brien R, Selby E, Joshi S, Subramanian V, Kadaba M, Schroeder K, Godson S, Comley K, Vollmer Sebastian J, Mateen BA (2020). Digital health management during and beyond the COVID-19 pandemic: opportunities, barriers, and recommendations. JMIR Ment Health.

[13] Lean M, Fornells-Ambrojo M, Milton A, Lloyd-Evans B, Harrison-Stewart B, Yesufu-Udechuku A, Kendall T, Johnson S (2019). Self-management interventions for people with severe mental illness: systematic review and meta-analysis. Br J Psychiatry.

[14] Edenfield TM, Saeed SA (2012). An update on mindfulness meditation as a self-help treatment for anxiety and depression. PRBM.

[15] Keng S, Smoski MJ, Robins CJ (2011). Effects of mindfulness on psychological health: a review of empirical studies. Clin Psychol Rev.

[16] Paluska SA, Schwenk TL (2000). Physical activity and mental health: current concepts. Sports Med.

[17] Beaulac Julie, Carlson AnnaMarie, Boyd R Jamie (2011). Counseling on physical activity to promote mental health: practical guidelines for family physicians. Can Fam Physician.

[18] Saxena S, Van Ommeren M, Tang KC, Armstrong TP (2009). Mental health benefits of physical activity. J Ment Health.

[19] (1999). Physical activity and health: a report of the Surgeon General. US Centers for Disease Control and Prevention.

[20] Hall G, Laddu DR, Phillips SA, Lavie CJ, Arena R (2021). A tale of two pandemics: how will COVID-19 and global trends in physical inactivity and sedentary behavior affect one another?. Prog Cardiovasc Dis.

[21] Burtscher J, Burtscher M, Millet GP (2020). (Indoor) isolation, stress, and physical inactivity: vicious circles accelerated by COVID-19?. Scand J Med Sci Sports.

[22] Carmody J, Baer RA (2008). Relationships between mindfulness practice and levels of mindfulness, medical and psychological symptoms and well-being in a mindfulness-based stress reduction program. J Behav Med.

[23] Lacaille J, Sadikaj G, Nishioka M, Carrière Kimberly, Flanders J, Knäuper Bärbel (2018). Daily Mindful Responding Mediates the Effect of Meditation Practice on Stress and Mood: The Role of Practice Duration and Adherence. J Clin Psychol.

[24] Wood W (2017). Habit in Personality and Social Psychology. Pers Soc Psychol Rev.

[25] Wood W, Rünger Dennis (2016). Psychology of habit. Annu Rev Psychol.

[26] Verplanken B, Orbell S (2003). Reflections on past behavior: a self‐report index of habit strength. J Appl Soc Psychol.

[27] Behan C (2020). The benefits of meditation and mindfulness practices during times of crisis such as COVID-19. Ir J Psychol Med.

[28] Torous J, Jän Myrick Keris, Rauseo-Ricupero N, Firth J (2020). Digital mental health and COVID-19: using technology today to accelerate the curve on access and quality tomorrow. JMIR Ment Health.

[29] Zhou X, Snoswell CL, Harding LE, Bambling M, Edirippulige S, Bai X, Smith AC (2020). The role of telehealth in reducing the mental health burden from COVID-19. Telemed J E Health.

[30] Huberty J, Green J, Glissmann C, Larkey L, Puzia M, Lee C (2019). Efficacy of the mindfulness meditation mobile app "Calm" to reduce stress among college students: randomized controlled trial. JMIR mHealth uHealth.

[31] Yang E, Schamber E, Meyer RML, Gold JI (2018). Happier healers: randomized controlled trial of mobile mindfulness for stress management. J Altern Complement Med.

[32] Economides M, Martman J, Bell MJ, Sanderson B (2018). Improvements in stress, affect, and irritability following brief use of a mindfulness-based smartphone app: a randomized controlled trial. Mindfulness (N Y).

[33] Puzia ME, Huberty J, Eckert R, Larkey L, Mesa R (2020). Associations between global mental health and response to an app-based meditation intervention in myeloproliferative neoplasm patients. Integr Cancer Ther.

[34] Huberty J, Eckert R, Larkey L, Kurka J, Rodríguez De Jesús Sue A, Yoo W, Mesa R (2019). Smartphone-based meditation for myeloproliferative neoplasm patients: feasibility study to inform future trials. JMIR Form Res.

[35] Calm COVID-19 survey. OSF.

[36] Form: CDC COVID-19 community survey question bank (DRAFT). US National Library of Medicine.

[37] Vartti A, Oenema A, Schreck M, Uutela A, de Zwart O, Brug J, Aro AR (2009). SARS knowledge, perceptions, and behaviors: a comparison between Finns and the Dutch during the SARS outbreak in 2003. Int J Behav Med.

[38] (2020). ZIP code data. US General Services Administration.

[39] Census Regions and Divisions of the United States. US Census Bureau.

[40] csse_covid_19_daily_reports_us. GitHub.

[41] Gardner B, Abraham C, Lally P, de Bruijn G (2012). Towards parsimony in habit measurement: testing the convergent and predictive validity of an automaticity subscale of the Self-Report Habit Index. Int J Behav Nutr Phys Act.

[42] Cohen S, Kamarck T, Mermelstein R (1983). A global measure of perceived stress. J Health Soc Behav.

[43] Cohen S, Spacapan S, Oskamp S (1988). Perceived stress in a probability sample of the United States. The Claremont Symposium on Applied Social Psychology. The Social Psychology of Health.

[44] Lee E (2012). Review of the psychometric evidence of the perceived stress scale. Asian Nurs Res (Korean Soc Nurs Sci).

[45] Hosey MM, Leoutsakos JS, Li X, Dinglas VD, Bienvenu OJ, Parker AM, Hopkins RO, Needham DM, Neufeld KJ (2019). Screening for posttraumatic stress disorder in ARDS survivors: validation of the Impact of Event Scale-6 (IES-6). Crit Care.

[46] Zigmond AS, Snaith RP (1983). The hospital anxiety and depression scale. Acta Psychiatr Scand.

[47] The R Project for statistical computing.

[48] Banerjee M, Cavanagh K, Strauss C (2018). Barriers to mindfulness: a path analytic model exploring the role of rumination and worry in predicting psychological and physical engagement in an online mindfulness-based intervention. Mindfulness (N Y).

[49] Stanton R, To QG, Khalesi S, Williams SL, Alley SJ, Thwaite TL, Fenning AS, Vandelanotte C (2020). Depression, anxiety and stress during COVID-19: associations with changes in physical activity, sleep, tobacco and alcohol use in Australian adults. Int J Environ Res Public Health.

[50] Schuch Felipe B, Bulzing Rugero A, Meyer Jacob, Vancampfort Davy, Firth Joseph, Stubbs Brendon, Grabovac Igor, Willeit Peter, Tavares Vagner Deuel O, Calegaro Vitor C, Deenik Jeroen, López-Sánchez Guillermo F, Veronese Nicola, Caperchione Cristina M, Sadarangani Kabir P, Abufaraj Mohammad, Tully Mark A, Smith Lee (2020). Associations of moderate to vigorous physical activity and sedentary behavior with depressive and anxiety symptoms in self-isolating people during the COVID-19 pandemic: a cross-sectional survey in Brazil. Psychiatry Res.

[51] Constandt B, Thibaut E, De Bosscher V, Scheerder J, Ricour M, Willem A (2020). Exercising in times of lockdown: an analysis of the impact of COVID-19 on levels and patterns of exercise among adults in Belgium. Int J Environ Res Public Health.

[52] Ammar Achraf, Brach Michael, Trabelsi Khaled, Chtourou Hamdi, Boukhris Omar, Masmoudi Liwa, Bouaziz Bassem, Bentlage Ellen, How Daniella, Ahmed Mona, Müller Patrick, Müller Notger, Aloui Asma, Hammouda Omar, Paineiras-Domingos Laisa Liane, Braakman-Jansen Annemarie, Wrede Christian, Bastoni Sofia, Pernambuco Carlos Soares, Mataruna Leonardo, Taheri Morteza, Irandoust Khadijeh, Khacharem Aïmen, Bragazzi Nicola L, Chamari Karim, Glenn Jordan M, Bott Nicholas T, Gargouri Faiez, Chaari Lotfi, Batatia Hadj, Ali Gamal Mohamed, Abdelkarim Osama, Jarraya Mohamed, Abed Kais El, Souissi Nizar, Van Gemert-Pijnen Lisette, Riemann Bryan L, Riemann Laurel, Moalla Wassim, Gómez-Raja Jonathan, Epstein Monique, Sanderman Robbert, Schulz Sebastian Vw, Jerg Achim, Al-Horani Ramzi, Mansi Taiysir, Jmail Mohamed, Barbosa Fernando, Ferreira-Santos Fernando, Šimunič Boštjan, Pišot Rado, Gaggioli Andrea, Bailey Stephen J, Steinacker Jürgen M, Driss Tarak, Hoekelmann Anita (2020). Effects of COVID-19 home confinement on eating behaviour and physical activity: results of the ECLB-COVID19 international online survey. Nutrients.

[53] Almandoz Jaime P, Xie Luyu, Schellinger Jeffrey N, Mathew Matthew Sunil, Gazda Chellse, Ofori Ashley, Kukreja Sachin, Messiah Sarah E (2020). Impact of COVID-19 stay-at-home orders on weight-related behaviours among patients with obesity. Clin Obes.

[54] Shechter A, Diaz F, Moise N, Anstey DE, Ye S, Agarwal S, Birk JL, Brodie D, Cannone DE, Chang B, Claassen J, Cornelius T, Derby L, Dong M, Givens RC, Hochman B, Homma S, Kronish IM, Lee SA, Manzano W, Mayer LE, McMurry CL, Moitra V, Pham P, Rabbani L, Rivera RR, Schwartz A, Schwartz JE, Shapiro PA, Shaw K, Sullivan AM, Vose C, Wasson L, Edmondson D, Abdalla M (2020). Psychological distress, coping behaviors, and preferences for support among New York healthcare workers during the COVID-19 pandemic. Gen Hosp Psychiatry.

[55] Nelson Benjamin W, Pettitt Adam, Flannery Jessica E, Allen Nicholas B (2020). Rapid assessment of psychological and epidemiological correlates of COVID-19 concern, financial strain, and health-related behavior change in a large online sample. PLoS One.

[56] Cheval Boris, Sivaramakrishnan Hamsini, Maltagliati Silvio, Fessler Layan, Forestier Cyril, Sarrazin Philippe, Orsholits Dan, Chalabaev Aïna, Sander David, Ntoumanis Nikos, Boisgontier Matthieu P (2021). Relationships between changes in self-reported physical activity, sedentary behaviour and health during the coronavirus (COVID-19) pandemic in France and Switzerland. J Sports Sci.

[57] COVID-19: coping with stress. US Centers for Disease Control and Prevention.

[58] Boyd JE, Lanius RA, McKinnon MC (2018). Mindfulness-based treatments for posttraumatic stress disorder: a review of the treatment literature and neurobiological evidence. J Psychiatry Neurosci.

[59] Hegberg NJ, Hayes JP, Hayes SM (2019). Exercise intervention in PTSD: a narrative review and rationale for implementation. Front Psychiatry.

[60] Penedo FJ, Dahn JR (2005). Exercise and well-being: a review of mental and physical health benefits associated with physical activity. Curr Opin Psychiatry.

[61] Rebar AL, Stanton R, Geard D, Short C, Duncan MJ, Vandelanotte C (2015). A meta-meta-analysis of the effect of physical activity on depression and anxiety in non-clinical adult populations. Health Psychol Rev.

[62] Warburton DER, Nicol CW, Bredin SSD (2006). Health benefits of physical activity: the evidence. CMAJ.

[63] Nieman DC, Wentz LM (2019). The compelling link between physical activity and the body's defense system. J Sport Health Sci.

[64] Morey JN, Boggero IA, Scott AB, Segerstrom SC (2015). Current directions in stress and human immune function. Curr Opin Psychol.

[65] Mani M, Kavanagh DJ, Hides L, Stoyanov SR (2015). Review and evaluation of mindfulness-based iPhone apps. JMIR mHealth uHealth.

[66] Plaza I, Demarzo MMP, Herrera-Mercadal P, García-Campayo Javier (2013). Mindfulness-based mobile applications: literature review and analysis of current features. JMIR mHealth uHealth.

[67] Fanning J, Mullen SP, McAuley E (2012). Increasing physical activity with mobile devices: a meta-analysis. J Med Internet Res.

