# Supplementary material for: The Effect of Meditation and Physical Activity on the Mental Health Impact of COVID-19–Related Stress and Attention to News Among Mobile App Users in the United States: Cross-sectional Survey
Source: JMIR Ment Health. 2021 Apr 13;8(4):e28479. doi: 10.2196/28479 (PMC8045775; doi:10.2196/28479)
Supplement: Multimedia Appendix 1 [file mental_v8i4e28479_app1.docx]

| **Table S1. Mediating effect of health behavior on depression symptoms** | | | | |
| --- | --- | --- | --- | --- |
|  | HADS Depression Score, Coefficient (SE) | | | |
|  | (1) | (2) | (3) | (4) |
| **COVID-19 worry – total effect** | 0.51^**^ (0.02) |  |  |  |
| **COVID-19 worry – indirect effects** |  | 0.49^**^ (0.02) | 0.51^**^ (0.02) | 0.50^**^ (0.02) |
| Frequency of physical activity |  | -0.13^**^ (0.02) |  |  |
| Stopped meditating |  |  | 1.16^**^ (0.22) |  |
| Strength of meditation habit |  |  |  | -0.06^**^ (0.01) |
| **COVID-19 attention – total effect** | 0.78^**^ (0.11) |  |  |  |
| **COVID-19 attention – indirect effects** |  | 0.75^**^ (0.11) | 0.70^**^ (0.12) | 0.71^**^ (0.12) |
| Frequency of physical activity |  | -0.17^**^ (0.02) |  |  |
| Stopped meditating |  |  | 1.25^**^ (0.23) |  |
| Strength of meditation habit |  |  |  | -0.07^**^ (0.01) |
| **Stress from social distancing – total effect** | 2.45^**^ (0.10) |  |  |  |
| **Stress from social distancing – indirect effects** |  | 2.39^**^ (0.10) | 2.43^**^ (0.11) | 2.41^**^ (0.11) |
| Frequency of physical activity |  | -0.15^**^ (0.02) |  |  |
| Stopped meditating |  |  | 1.13^**^ (0.22) |  |
| Strength of meditation habit |  |  |  | -0.06^**^ (0.01) |
| ^*^*P<*.10, ^**^*P<*.05,^***^*P<*0.01 | | | | |
| *Note.* Age, racial minority status, female, Hispanic, high-school education only, undergraduate education, income <$80,000, income $81,000-$100,000, unemployed, underlying medical condition, and living in state with high COVID-19 prevalence were included as covariates in all models. | | | | |

The relationship between worry about COVID-19 and depression symptoms was significantly mediated by changes in physical activity (*P<*.001) and strength of meditation habit (*P<*.001), but not by stopping meditation (*P*=.08).

The relationship between attention to COVID-19 news and updates and depression symptoms was significantly mediated by changes in physical activity (*P*=.01), stopping meditation (*P*=.01), but not strength of meditation habit (*P*=.11).

The relationship between stress caused by COVID-19 social distancing recommendations and depression symptoms was significantly mediated by changes in physical activity (*P*=.002), stopping meditation (*P*=.01), and strength of meditation habit (*P*<.001).

| **Table S2. Mediating effect of health behavior on anxiety symptoms** | | | | |
| --- | --- | --- | --- | --- |
|  | HADS Anxiety Score, Coefficient (SE) | | | |
|  | (1) | (2) | (3) | (4) |
| **COVID-19 worry – total effect** | 0.30^**^ (0.02) |  |  |  |
| **COVID-19 worry – indirect effects** |  | 0.29^**^ (0.02) | 0.30^**^ (0.02) | 0.30^**^ (0.02) |
| Frequency of physical activity |  | -0.25^**^ (0.02) |  |  |
| Stopped meditating |  |  | 1.78^**^ (0.20) |  |
| Strength of meditation habit |  |  |  | -0.11^**^ (0.01) |
| **COVID-19 attention – total effect** | 0.42^**^ (0.10) |  |  |  |
| **COVID-19 attention – indirect effects** |  | 0.38^**^ (0.10) | 0.32^**^ (0.11) | 0.34^**^ (0.11) |
| Frequency of physical activity |  | -0.27^**^ (0.02) |  |  |
| Stopped meditating |  |  | 1.83^**^ (0.21) |  |
| Strength of meditation habit |  |  |  | -0.12^**^ (0.01) |
| **Stress from social distancing – total effect** | 1.96^**^ (0.09) |  |  |  |
| **Stress from social distancing – indirect effects** |  | 1.84^**^ (0.09) | 1.93^**^ (0.10) | 1.90^**^ (0.10) |
| Frequency of physical activity |  | -0.26^**^ (0.02) |  |  |
| Stopped meditating |  |  | 1.73^**^ (0.20) |  |
| Strength of meditation habit |  |  |  | -0.11^**^ (0.01) |
| ^†^*P<*.10, ^*^*P<*.05, ^**^*P<*0.01 | | | | |
| *Note.* Age, racial minority status, female, Hispanic, high-school education only, undergraduate education, income <$80,000, income $81,000-$100,000, unemployed, underlying medical condition, and living in state with high COVID-19 prevalence were included as covariates in all models. | | | | |

The relationship between worry about COVID-19 and anxiety was significantly mediated by changes in physical activity (*P<*.001) and strength of meditation habit (*P=*.002), but not by stopping meditation (*P*=.08).

The relationship between attention to COVID-19 news and updates and anxiety was significantly mediated by changes in physical activity (*P*=.01), stopping meditation (*P*=.01), but not strength of meditation habit (*P*=.14).

The relationship between stress caused by COVID-19 social distancing recommendations and anxiety was significantly mediated by changes in physical activity (*P*=.01), stopping meditation (*P*=.01), and strength of meditation habit (*P*<.001).
